# Short Onset and Enhanced Analgesia Following Nasal Administration of Non-Controlled Drugs in Nanovesicular Systems

**DOI:** 10.3390/pharmaceutics13070978

**Published:** 2021-06-28

**Authors:** Elka Touitou, Hiba Natsheh, Shatha Boukeileh, Rania Awad

**Affiliations:** The Institute for Drug Research, School of Pharmacy, Faculty of Medicine, The Hebrew University of Jerusalem, P.O. Box 12272, Jerusalem 9112002, Israel; hiba.natsheh@mail.huji.ac.il (H.N.); shathab@ekmd.huji.ac.il (S.B.); rania.awad@mail.huji.ac.il (R.A.)

**Keywords:** analgesia short onset, phospholipid soft vesicles, nasal administration, nanovesicular carrier, pain management, tramadol HCl, ketoprofen, butorphanol tartrate

## Abstract

Nasal nanovesicular delivery systems (NVS) containing the noncontrolled analgesic drugs Ketoprofen, Butorphanol or Tramadol, incorporated in a phospholipid nanovesicular carrier, were designed and investigated. The systems were first characterized for their physicochemical properties. Due to their composition, comprising propylene glycol as a lipid bilayers fluidizer, these systems contain soft vesicles. Pharmacokinetic profiles of Tramadol in plasma and brain and of Ketoprofen in plasma were also assessed. The analgesic effect of each of the three tested drugs was evaluated in the acetic acid mice model for pain. One important result obtained in this work is that the concentration of Tramadol in rats’ plasma and brain increased rapidly after administration, reaching a peak value 10 min after administration with a C_max_ of 2 to 5 folds greater than that for the oral or nasal non-vesicular treatments, respectively. In the case of Ketoprofen, the peak of the drug level in plasma was measured 10 min post nasal administration in NVS. The C_max_ was three-fold higher relative to oral administration of this drug. In the experiment testing analgesia, a rapid and improved analgesia was observed for the tested drugs when delivered nasally in the nanocarrier. On the other hand, a weaker analgesic effect was observed for oral and nasal control systems. This new approach suggests that nasal delivery of non-controlled drugs in soft nanovesicles may open the way for better and noninvasive treatment of severe pain.

## 1. Introduction

Short onset of analgesia is crucial for the effective management of moderate and severe pain, such as cancer pain. Parenteral administration of analgesics provides a rapid onset of pain relief, but this method of drug administration is painful, inconvenient and requires medical service assistance [1,2]. Thus, there is a need for investigating other approaches for rapid and effective pain control.

Orally administrated immediate release controlled opioids, such as Morphine, are usually given in such cases. However, the onset of action of these medications may take up to 20–30 min, with a peak of analgesia after one hour or more [2,3]. In addition, oral drug administration can be associated with slow onset of action, extensive hepatic first pass effect and serious GI problems [4].

The nasal pathway is noninvasive and painless. It avoids intestinal and hepatic metabolism, can be utilized for delivery of therapeutic agents to the systemic circulation and to the brain [5] and has good patient compliance [6], allowing self-administration and enabling home treatment [7]. Nasal administration of opioids including Morphine and Fentanyl has been investigated in number of preclinical and clinical studies [2,8,9,10,11]. However, the use of controlled opioids is usually associated with many side effects, including tolerance and addiction [12].

The aim of this work was to design and investigate novel nasal delivery systems containing non-controlled central- and systemic-acting analgesics.

Due to the permeability barrier in the nasal mucosa, the delivery of the majority of such active agents is poor when administrated in aqueous solution [6]. In this sense, several approaches have been considered to overcome the limitations of this route and improve the analgesic effect of non-controlled central- and systemic-acting analgesic agents. These include the use of absorption enhancers formulated in various pharmaceutical forms, such as in situ and chitosan hydrogels, mucoadhesive nanostructured lipid carriers and chitosan nanoparticles [13,14,15,16]. However, some of these approaches may lead to nasal toxicity and leaching of membrane components including proteins and enzymes, which may cause local irritation to the nasal mucosa [17].

Our group has pioneered the design of nasal nanocarriers based on phospholipid soft vesicles for rapid and enhanced effect of active molecules [18,19,20,21,22,23,24]. In a recent review, we have emphasized that phospholipid soft nanovesicles are generated in the presence of alcohols [25]. The first nasal carrier, containing nanovesicles with bilayers altered by ethanol, was introduced by Touitou et al. [18]. The fluidity of the vesicle bilayers was confirmed by results of differential scanning calorimetry (DSC) measurements indicating a difference of more than 16 °C in the transition temperature (T_m_) of the phospholipid in these vesicles compared to classic liposomes. This carrier has shown an ability to enhance drug delivery to the brain and the systemic circulation. In addition, improved effect of various active molecules such as Buspirone, Tramadol HCl and Glatiramer acetate was achieved by means of nasal administration in this carrier without causing irritation or toxicity to the nasal mucosa.

Recently, we have studied another safe nasal carrier containing phospholipid soft vesicles, the Phospholipid Magnesome, for direct nose to brain drug delivery [22,23]. This carrier is composed of phospholipid vesicles containing magnesium. Electronic microscopy and dynamic light scattering indicated the presence of multilamellar spherical vesicles in the nanoscale range. DSC data, revealing a T_m_ value of −7 °C for the phospholipid in the Magnesome vesicles vs. +7 °C for liposomes, confirms fluidization of the phospholipid lamellae in these vesicles.

Different from other vesicular compositions, the above nasal carriers are prepared at room temperature using a simple mixing method.

In the present work, nasal phospholipid delivery systems (NVS) containing soft vesicles and Ketoprofen, Butorphanol or Tramadol were designed and investigated. The physical characteristics of the systems were studied by several methods including electron microscopy, dynamic light scattering, entrapment efficiency and pH. Pharmacokinetic profiles of Tramadol in plasma and brain and of Ketoprofen in plasma were also assessed. The analgesic effect of these systems was evaluated in the acetic acid mice model for pain. The local safety on the nasal mucosa of the investigated carrier was examined following sub-chronic administration to rats.

## 2. Materials and Methods

### 2.1. Materials

Phospholipon 90 G (PL), a soy phospholipid, was obtained from Lipoid-Phospholipid GMBH, Köln, Germany. Ketoprofen (KET) of 99% purity, was purchased from Sigma-Aldrich (Rehovot, Israel). Butorphanol tartrate (BUT) was obtained from Czech industries s.r.o. (Opava, Czech Republic). Tramadol HCl (TRA) was purchased from Chemagis (Bnei Brak, Israel). Rhodamine 6G (R6G) of ~95% purity, Fluorescein isothiocyanate (FITC) of ≥95% purity, Methylcellulose 1200–1800 cP, 2% in water (20 °C) and Sodium alginate (from brown algae) were purchased from Sigma-Aldrich (Israel). All other used materials were of pharmaceutical or analytical grade.

### 2.2. Animals

All the in vivo experiments were carried out in accordance with institutional guidelines for animal care, by protocols approved by the Animal Ethical Care Committee of the Hebrew University of Jerusalem (MD-09-11949-3, 11/08/2009; MD-11-12825-4, 17/07/2011; MD-17-15076-5, 25/02/2017).

The pharmacokinetic experiments were performed on adult male SD/Hsd rats weighing 300–350 g. The antinociceptive effect of the tested systems was carried out on female C57BL/6 mice (Harlan, Israel) weighing 19–23 g.

The animals were housed under standard conditions of light and temperature, in plastic cages, in the specific-pathogen free (SPF) unit of the Faculty of Medicine animal housing facility at the Hebrew University. Animals were housed under normal 12-h light/dark cycle and a temperature of 21–22 °C, with food and water freely available.

For nasal drug administration, the animals were briefly anesthetized with Isoflurane and held in an upright position to mimic the human position. The compositions were then applied equally into the two nasal nostrils by a micropipette at a total volume of 7–12 and 15–20 µL for mice and rats, respectively.

### 2.3. Preparation of Nasal Nanovesicular Carrier and Drug Containing NVS

The systems were prepared using Phospholipon 90G. The phospholipid (2–3% *w*/*w*) was dissolved in propylene glycol (20–30% *w*/*w*) with continuous mixing at 500 rpm using an overhead stirrer (RZR-2000, Heidolph^®^, Schwabach, Germany). Then, double distilled water (DDW) was slowly added with mixing. The drug was dissolved in DDW or added to the final composition with mixing. The pH was then adjusted if required. The compositions were prepared at room temperature. The preparations were used in all experiments without any separation of the vesicles from the system. A number of systems containing various concentrations of the drug (Tramadol 0.9 and 10% *w*/*w*, Ketoprofen 3 and 20% *w*/*w* and Butorphanol 0.04 and 1% *w*/*w*) were prepared. Selected compositions, presented further in a Table in the results section, were characterized.

The investigated analgesic drugs and their properties are given in Table 1.

For comparative studies, the following compositions were used: oral Ketoprofen suspension was prepared by dispersing the drug in a 2% methyl cellulose aqueous suspension. For Butorphanol, a water solution for nasal administration was prepared by dissolving the drug in water. Tramadol–water solution for oral administration (PO) was obtained by dissolving the drug in water, and the nasal non-vesicular system (NV), was prepared by dissolving Tramadol in 0.6% alginate aqueous solution. The preparation of all the above formulations was carried out at room temperature and by mixing with an overhead stirrer at 500 rpm.

### 2.4. Physicochemical Characterization of Nasal Nanovesicular Systems

#### 2.4.1. Visualization of Vesicles Structure by Transmission Electron Microscope (TEM)

The nasal nanovesicular carrier and NVS containing Butorphanol were visualized by Transmission Electron Microscope (TEM) (Philips TECHNAI CM 120 electron microscope, Eindhoven, The Netherlands). Samples were applied on Carbon Film Grid, negatively stained with 1% aqueous solution of phosphotungstic acid (PTA) for 30 s and viewed under the microscope at a 37–135 k-fold enlargement.

#### 2.4.2. Accumulation of Molecules within the Carrier Vesicles Visualized by Confocal Laser Scanning Microscopy (CLSM)

Systems containing Rhodamine 6G (R6G) or Fluorescein isothiocyanate (FITC) at concentrations of 0.1 and 0.5% *w*/*w*, respectively, were examined. Samples of 20 µL were applied on a glass slide (76 × 76 mm, Marienfeld Microscope Slides, Baden-Württemberg, Germany), covered with 22 × 22 mm plastic coverslip and examined using CLSM (Olympus Fluoview 300, Tokyo, Japan), with an air plan 10 × 0.40 NA objective lens, at an excitation wavelength of 543 nm for R6G and 488 nm for FITC.

#### 2.4.3. Measurement of Vesicles Size Distribution

Mean size distribution of vesicles in the carrier and NVS containing analgesic molecules was measured by dynamic light scattering (DLS) using a computerized Malvern Zetasizer-nano System (ZEN 3600, Malvern Instruments, Worcestershire, UK). Systems were diluted with hydro-glycolic solution at a 1:500 ratio. Sets of triplicates were prepared. Each batch was tested in 3 measurements at 25 °C. The duration and the set position of each measurement were automatically fixed by the apparatus. The test was performed at an angle of 173° to measure the size distribution by intensity.

#### 2.4.4. pH Measurements

Two batches of diluted samples (1:5 with DDW) were measured for each system using the Seven Easy pH meter (Seven Easy pH meter, Mettler Toledo, Greifensee, Switzerland).

### 2.5. Quantification of Analgesic Drugs in Rats’ Plasma and Brain Following Administration in the Nanovesicular System

Pharmacokinetic profiles were assessed for Ketoprofen in plasma and for Tramadol in plasma and brain. Rats were chosen for this experiment to ensure collection of sufficient blood samples for HPLC analysis.

#### 2.5.1. Measurement of Ketoprofen Concentration in Plasma

Ketoprofen was administered nasally to rats from the Ketoprofen nasal nanovesicular system (KET-NVS) at a dose of 14 mg/kg and compared to oral preparation (KET-PO), each containing 20% drug. Blood samples (~500 µL) were collected from rats’ tails at 10, 30, 60, 120, 180, 240 and 300 min post drug administration.

#### 2.5.2. Measurement of Tramadol Levels in Brain and Plasma

The concentration of Tramadol in rat brain tissue and plasma was measured at various time points post nasal drug administration at a dose of 7 mg/kg in NVS, non-vesicular carrier (NV) or Oral preparation (PO), each containing 10% *w*/*w* drug.

Eighty rats were divided into groups of five animals for testing the four treatments at five time points. Blood samples were taken 15, 30, 60, 120 or 240 min after treatment, then the animals were sacrificed, and the brains were collected.

### 2.6. Drug Extraction and Analytical Tests

#### 2.6.1. Extraction of Ketoprofen from Plasma

Blood samples were centrifuged at 3 k rpm for 10 min at 25 °C, then plasma (150 µL) was transferred into 1.5 mL Safe-Lock Eppendorf Tubes^®^ (Eppendorf AG, Hamburg, Germany). Plasma samples were kept at −20 °C until analysis. On the day of analysis, the plasma samples, 100 µL, were thawed and 300 µL of acetonitrile were added and mixed by vortex Genie^®^ (Scientific Industries, Bohemia, NY, USA) for 3 min at level 10. Then 300 µL of acetate buffer 0.05 M, pH 5, were added and mixed by vortex for an additional 1 min at the same level. All samples were centrifuged for 5 min at 14 k rpm and 25 °C. The supernatants were filtered through a Bulk GHP Acrodisc^®^ 13 mm syringe filter with 0.45 um GHP membrane (Pall Corporation, Washington, NY, USA), and transferred into pre-labeled auto injector vials before being injected into HPLC-UV.

#### 2.6.2. HPLC Assay of Ketoprofen

Ketoprofen levels in rats’ plasma were quantified by reverse phase HPLC (Hitachi 7000 HPLC, Tokyo, Japan) equipped with UV variable detector and HSM computerized analysis program. Detection was carried out at 254 nm using LiChrospher^®^ C18, 100 mm × 250 mm, 5 µm column with a mobile phase of acetonitrile: acetate buffer 0.05 M pH 5.0 (40:60, *v*/*v*) at a flow rate of 1.0 mL/min. The calibration data were fitted to a straight line with an R^2^ value of 0.9999. Other measured validation parameters were precision (2.26%), accuracy (0.26%) and recovery (114.4%). The lower limit of detection (LOD) and the lower limit of quantification (LOQ) were 0.069 and 0.211 µg/mL, respectively.

#### 2.6.3. Extraction of Tramadol from Plasma and Brain

Tramadol was quantified in plasma and brain tissue by a liquid–liquid extraction method used by Touitou et al. [21]. Blood samples were centrifuged at 3 k rpm for 10 min at room temperature, to separate the plasma, and the brains were homogenized with 2 mL/g water. The samples were alkalinized with 0.1 M NaOH to pH ~ 11. Five milliliters of co-solvent mixture (Ethyl Acetate: Hexane 1:4 *v*/*v*) were added and the samples were mixed well by vortex. The brain mixtures undergone additional mixing by Labquake Shaker (Lab Industries, Los Angeles, CA, USA) for 20 min. Then, all the samples were centrifuged for 30 min at 4 k rpm at 4 °C. The supernatants were collected and evaporated under vacuum at 35 °C until completely dried (~45 min). The dried residues were reconstituted with the HPLC mobile phase and centrifuged at 14 k rpm for 10 min at RT. Final supernatants were filtered through the Acrodisc^®^ syringe filters, GHP membrane, diam. 13 mm, pore size 0.45 μm (Pall Corporation, Washington, NY, USA) and injected to HPLC.

#### 2.6.4. Tramadol HPLC Assay

Tramadol quantification in all samples was performed by a method previously published by Duchi et al. and Touitou et al. [19,21] using an HPLC apparatus (Hitachi 7000 HPLC, Tokyo, Japan) equipped with UV variable detector and an HSM computerized analysis program. The separation and detection of Tramadol were carried out at 220 nm using a mobile phase composed of acetonitrile: phosphate buffer 0.01 M containing 0.1% Triethylamine (22:78), adjusted to pH 3 with orthophosphoric acid through the Apollo^®^ C18 5u RP-C18 (5 µm particles size, 25 cm × 4 mm) I.D column at a 1 mL/min flow rate. The calibration data were fit to a straight line with an R^2^ value of 0.9901. The drug recovery assays for brain tissue and plasma yielded results of 98.2 and 52.1%, respectively. The LOD and the LOQ were 0.051 and 0.153 μg/mL, respectively.

### 2.7. Evaluation of Antinociceptive Effect in Animal Model for Pain of Ketoprofen, Butorphanol Tartrate and Tramadol Nasal Nanovesicular Systems

In this set of experiments, the effect of the new nanocarrier on the antinociceptive effect of the investigated drugs was measured using the acetic acid induced animal model for pain. In this model, an intraperitoneal (i.p.) injection of a weak solution of 0.6% acetic acid (10 mL/kg) induces a nociceptive stereotyped behavior (writhing) that mimics acute visceral pain. We have chosen mice for this experiment based on several studies using mice for an animal pain model [19,21,23,26,27]. The injection of acetic acid generates writhes, which are indicated by body elongation with stretching of at least one hind limb. The analgesic effect of the tested drugs, given under Isoflurane^®^ anesthesia (up to one minute), is evaluated by counting the number of writhes 5 min after acetic acid injection for a period of 10 min compared to untreated mice.

The analgesic effect of different treatments is expressed by the Maximum Possible Effect (MPE%), which is directly related to the efficiency of the treatment, and is calculated according to the following equation:

MPE% = [Mean of writhes in untreated control group − Mean of writhes in treated group]/[Mean of writhes in untreated control group] × 100

The antinociceptive effect of the various nanovesicular systems was tested. The administrated dose of each molecule, the drug concentration in the tested systems and the tested time points are presented in Table 2. For each tested system, an additional five mice served as an untreated control group with induced pain.

### 2.8. Local Safety Assessment

In this experiment, the safety of NVS on the nasal cavity was evaluated in rats by a method previously used by Duchi et al. [19]. In brief, ten female HSD rats (220–250 g) were divided into four treatment groups and one untreated control group (n = 3/group). Rats in the treatment groups received 15 μL NVS, Normal Saline nasal solution (NS) or Sodium Lauryl Sulfate (SLS) nasal solution into both nostrils twice a day for 7 days. At the end of the treatment period, animals were sacrificed and the nasal cavities were removed and fixed in 3.7% Formaldehyde in PBS. The nasal cavities were cut into serial sections of 7 μm thickness and stained with Hematoxylin and Eosin. The sections were examined by a professional histopathologist from the Authority for Animal Facilities, Hebrew University of Jerusalem, by Olympus light microscope BX43. Micrographs of the tested sections were taken by Olympus digital camera DP21 with Olympus cellSens Entry 1.13 software (Olympus, Tokyo, Japan) at 10× magnification. The local toxicity [28] was assessed by evaluating the histopathological alterations in different regions of the nasal cavity (cartilage and turbinate bone, lamina propria and submucosa, mucosal epithelium and lumen).

### 2.9. Data Analysis

The pharmacokinetic parameters used to analyze Tramadol concentration–time profiles in rats’ plasma and brain, and the Ketoprofen concentration–time profile in rats’ plasma, were: T_max_, C_max_, AUC_0–last_ and AUC_0–∞__._ The AUC _0–last_, and AUC_0–∞_ for each treatment group was considered the mean of the area under the curve for individual animals from 0 time to the last tested time point or from 0 time to infinity, respectively. Analysis of the pharmacokinetic data was carried out using the NCOMP program, a Windows-based computer program for noncompartmental analysis of pharmacokinetic data [29].

The relative bioavailability (F) of Tramadol in rats’ plasma and brain and of Ketoprofen in plasma from the nanovesicular carrier was calculated from noncompartmental parameters using the following equation:

F = [(AUC_0–last_ [NVS] × DOSE (PO or NV)]/[(AUC_0–last_ (PO or NV) × DOSE (NVS)] (1)

### 2.10. Statistical Analysis

The results are expressed as mean± SD or mean ± SEM. Statistical analysis of the pharmacokinetic data was performed using the Mann–Whitney two-tailed test (unpaired nonparametric test). The Mann–Whitney one-tailed test (unpaired nonparametric test) was used for the testing of data obtained in the antinociceptive effect evaluation of the drugs, with *p* < 0.05 considered significant and *p* < 0.01 very significant.

## 3. Results

### 3.1. Physicochemical Characterization of Carrier and Drug Loaded NVS

Selected systems and their composition were characterized (Table 3).

The presence of vesicles in the nanovesicular carrier and the system containing drug was examined by TEM.

**Figure 1:**
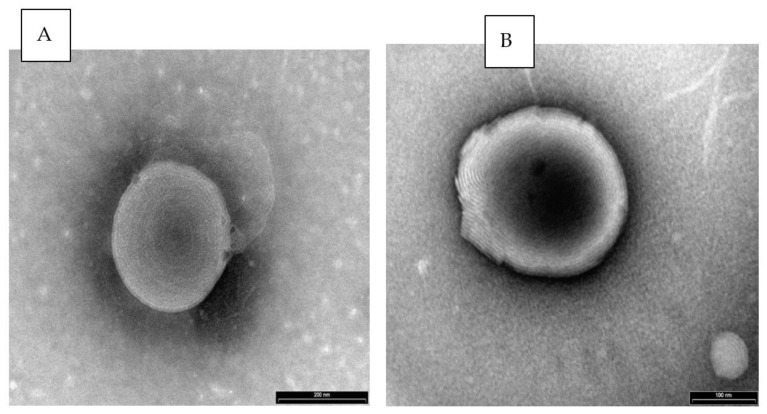
Representative TE micrograph (**A**) Nanovesicular carrier not containing drug ×135 k, bar 200 nm, and (**B**) NVS containing 1% Butorphanol tartrate, ×135 k bar 100 nm, Philips TECHNAI CM 120 electron microscope, Eindhoven, The Netherlands.

TE micrographs of samples negatively stained with PTA and observed under a Philips CM 120 TEM are given in Figure 1. The multilamellar vesicles in both the carrier and Butorphanol NVS can be clearly seen.

**Figure 2:**
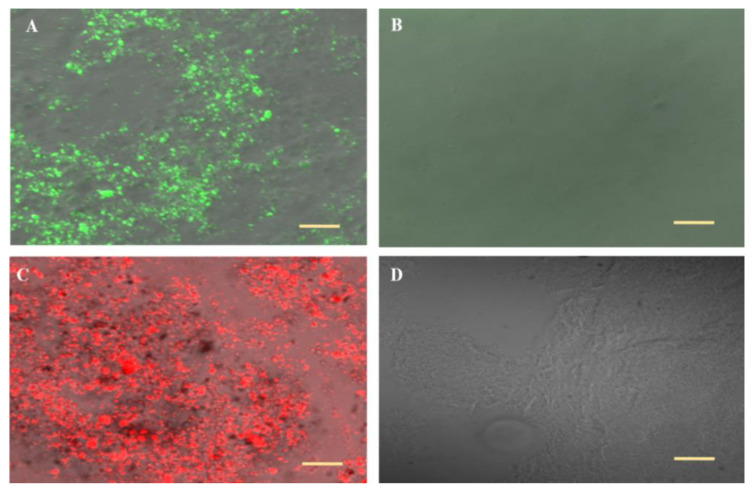
Representative CLS micrographs for (**A**) Nanovesicular carrier containing FITC, (**B**) a hydro-glycolic solution lacking the phospholipid containing FITC, (**C**) Nanovesicular carrier containing R6G and (**D**) a hydro-glycolic solution lacking the phospholipid containing R6G. Olympus Fluoview 300, Tokyo, Japan, Lens × 60 (Bar = 10 μm).

The ability of the vesicles to entrap various molecules can be seen in CLS micrographs (Figure 2A,C). The hydrophilic R6G and the lipophilic FITC are accumulated within the vesicles. Control systems lacking the phospholipid have not shown probe accumulation (Figure 2B,D). These data suggest that analgesic drugs with different properties can be incorporated in the new carrier vesicles.

The vesicles’ mean size distribution was measured for various systems. As an example, the size of the vesicles in KET-NVS, BUT-NVS and TRA-NVS were found to be 99.41 ± 5.74, 508.89 ± 70.23 and 453.1 ± 33.0 nm, respectively.

These phospholipid nanovesicular systems contain soft vesicles due to the presence of propylene glycol, a lipid bilayers fluidizer [23,25]. The pH values for KET-NVS, BUT-NVS and TRA-NVS were 5.54 ± 0.06, 5.79 ± 0.03 and 5.0 ± 0.04, respectively.

### 3.2. Pharmacokinetic Profiles Following Drugs Nasal Administration in NVS

#### 3.2.1. Ketoprofen Levels in Rats’ Plasma

The influence of the nasal nanovesicular carrier on the absorption parameters of the drug was evaluated by assessment of Ketoprofen concentration in the rats’ plasma following drug administration from nasal nanovesicular carrier as compared to oral preparation.

**Figure 3:**
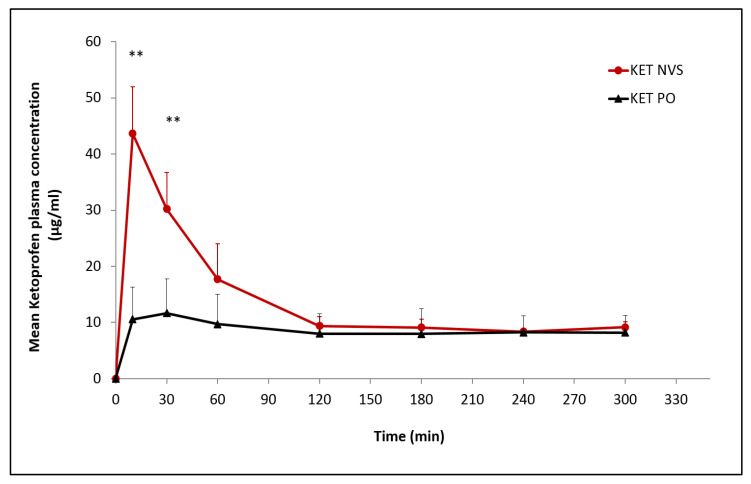
Pharmacokinetic profile of Ketoprofen in plasma following nasal administration of the drug nanovesicular system (KET-NVS), as compared to oral preparation (KET-PO), each at a drug dose of 14 mg/kg, (n, 5 for each group), (Mean ± SD). ** *p* < 0.01 KET-NVS vs. KET-PO at 10 min, and 30 min.

Ketoprofen levels were measured in the rats’ plasma starting 10 min after nasal and oral administration. Results show very significantly higher plasma concentrations detected during the first 30 min following Ketoprofen nasal administration (*p* < 0.01). C_max_ plasma values for nasal and oral administration were 43.65 ± 8.30 and 11.64 ± 6.11 µg/mL, respectively, at Tmax values of 10 and 30 min, respectively. The drug levels in the nasally treated group decreased at 30 min after reaching the C_max_, remaining superior to the PO group for 120 min, and then became comparable for the two groups until the end of the five-hour experiment. The calculated relative bioavailability was 1.56 (Figure 3, Table 4).

#### 3.2.2. Tramadol Levels in Rats’ Brain and Plasma

**Figure 4:**
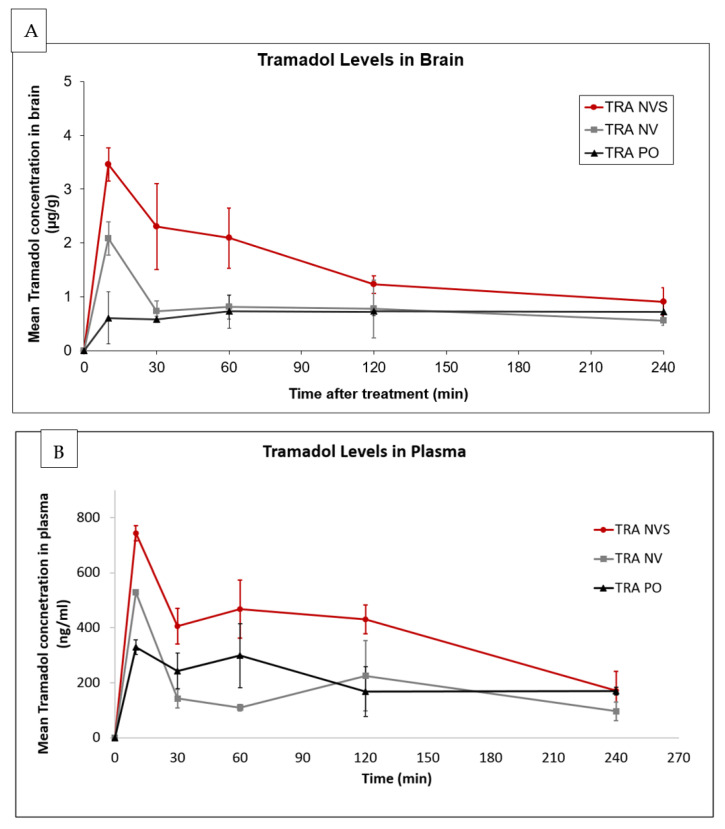
Pharmacokinetic profiles of Tramadol in (**A**) brain and (**B**) plasma following administration of the drug in the nasal nanovesicular system (TRA NVS) as compared to nasal non-vesicular system (TRA NV) and to oral administration (TRA PO), each at a drug dose of 7 mg/kg; mean ± SD; n, 5/group. (**A**) *p* < 0.01 for NVS vs. NV and 10, 30 and 60 min, and vs. PO at 10 min and 30 min; (**B**) *p* < 0.01 for NVS vs. NV and PO at 10–120 min.

Brain and plasma concentration vs. time profiles of the drug following nasal administration to male HSD rats at a dose of 7 mg/kg from NVS, nasal non-vesicular carrier (NV) and of tramadol oral aqueous solution (PO) are given in Figure 4.

Drug levels in brain tissue of 3.46 ± 0.31 µg/g were detected 10 min post nasal drug administration in NVS. At this time point, 60 and 17% lower levels (2.08 ± 0.31 and 0.60 ± 0.48 µg/g) were measured for the NV and PO treatment groups, respectively (Figure 4A). The C_max_ values were achieved at 10 min for the nasally treated groups (NVS and NV) and 60 min for the PO group. The drug levels decreased in the nasally treated groups after reaching the C_max_, in the NVS and NV groups, and then became stable starting from 120 and 30 for the NVS and NV groups, respectively, with a superior profile for the NVS group until the end of the experimental period. On the other hand, the orally treated group showed lower levels compared to the NVS-treated animals.

Plasma concertation values were higher by 1.5 and 5 folds for animals treated nasally with the nanosystem in comparison with those treated with the nasal non-vesicular system (NV) or by oral administration (PO), respectively. Up to two-folds higher plasma drug concentration was measured: 743.8 ± 27.7, 529.2 ± 9.5 and 329.5 ± 26.0 ng/mL at 10 min for the NVS, NV and PO groups, respectively. The level in the NVS group was maintained above the 400 ng/mL for 120 min. On the other hand, plasma drug levels in animals of the NV group decreased drastically to around 100 ng/mL, remaining at the low range until the end of the four-hour experiment.

The pharmacokinetic parameters in brain and plasma for Tramadol administrated in the three systems, analyzed using MCOMP software, are presented in Table 5.

These results suggest a rapid and efficient delivery of Tramadol to the brain and plasma by means of NVS. C_max_ and AUC values for the system in comparison to the other groups show improved delivery to the brain when the NVS is used. The lower bioavailability following drug nasal administration in the non-vesicular system emphasizes the important role of the phospholipid soft vesicles in enhancing the nasal delivery of Tramadol to the brain and its systemic circulation.

### 3.3. Analgesic Effect of Drugs Administered in the Nasal Nanovesicular Systems as Compared to Other Nasal and Oral Systems in Animal Model for Pain

The analgesic effect of Ketoprofen 30 min after nasal administration in the nanovesicular system was evaluated in an acetic acid pain-induced mice model as compared to oral administration of the drug and to untreated animals.

**Figure 5:**
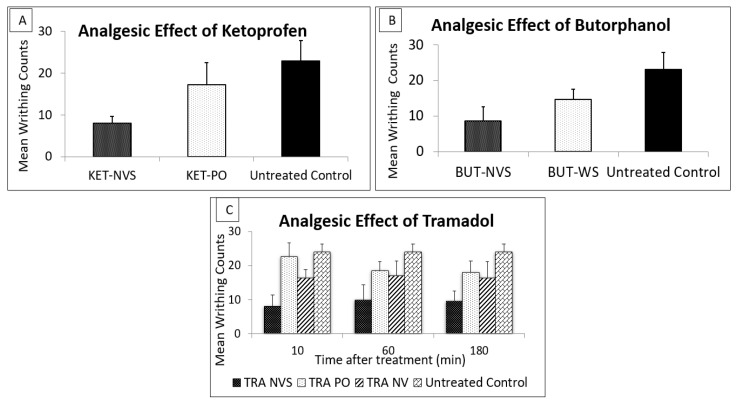
Mean writing counts in mice treated with (**A**) Ketoprofen nasal nanovesicular system (KET-NVS), vs. oral drug administration (KET-PO), each at a dose of 11 mg/kg, 30 min prior to i.p. injection of acetic acid and compared to untreated mice, (n, 4/each group). (**B**) Butorphanol nanovesicular system (BUT-NVS), vs. water solution (BUT-WS), each at a dose of 0.15 mg/kg, 10 min prior to i.p. injection of acetic acid and compared to untreated control mice, (n, 5/each group). (**C**) Tramadol at a dose of 5 mg/kg in Nasal Nanovesicular carrier (TRA NVS), Nasal non-vesicular system (TRA NV), Oral preparation (TRA PO) and untreated mice 10, 60 and 180 min prior to i.p. injection of acetic acid; (n, 5/each group), mean ± SD. *p* < 0.05 for KET-NVS-treated group vs. KET-PO. *p* < 0.01 for KET-NVS-treated group vs. untreated control. *p* < 0.05 for BUT NVS vs. BUT PO, *p* < 0.01 for BUT NVS vs. untreated. *p* < 0.05 for TRA NVS vs. TRA PO, and TRA NV and untreated at all time points.

Ketoprofen administered nasally at a dose of 11 mg/kg in the nasal nanovesicular carrier resulted in a significant reduction in the mean number of writhes (8.6 ± 1.63) as compared to oral administration (17.25 ± 5.32) and untreated animals (23 ± 4.85). The oral administration of Ketoprofen was not sufficient to produce a good analgesia, as there was no significant reduction in the number of writhes in comparison to the untreated animals (Figure 5A).

The calculated MPE of Ketoprofen delivered from the nasal nanovesicular system was 67.0%, which is significantly higher than the values obtained after oral administration (28.87%, *p* < 0.05). The improved analgesic effect of KET NVS is sustained by its superior pharmacokinetic plasma profile relative to oral administration.

Further, it was interesting to test the behavior of the Butorphanol NVS against the nasal aqueous spray of this drug currently used for management of acute pain. Ten minutes after drug administration, a very significant lower mean writhing count (8.6 ± 4.04) was measured in mice treated nasally with Butorphanol tartrate from the nanovesicular system in comparison to the untreated control mice (23 ± 4.85 writhes) or those treated with Butorphanol from the nasal water solution (14.6 ± 2.90 writhes) (Figure 5B). The mean MPE values were found to be 62.6 and 36.5% for the nanovesicular system and the water solution, respectively. These results indicate the ability of the nanosystem to improve the analgesic effect of Butorphanol nasal aqueous spray.

The analgesic effect of Tramadol at a dose of 5 mg/kg delivered nasally in NVS and in various control systems was evaluated in the acetic acid mouse model for pain. The results presented in Figure 5C and Table 6 show that Tramadol NVS led to a short onset of action (10 min) and more than 100% reduction in pain expression, from 24.3 ± 3.2 writhes in untreated animals to 8.9 ± 0.7 for the treatment with Tramadol in the new carrier. The analgesia was maintained through the entire period of testing (180 min). The weak analgesia and, consequently, the low MPE% in the animal groups treated nasally with the drug from the nonvesicular system (NV) or by oral administration (PO) was two to five folds lower than Tramadol in NVS.

These results, showing that drug delivery from NVS containing soft vesicles is 100% higher than the controls, sustain the role of this vesicular carrier in improving the analgesia efficiency of Tramadol.

### 3.4. Local Safety of NVS

The safety of the NVS System following sub-chronic treatment on the nasal cavity was assessed and compared to Normal Saline (NS) or sodium lauryl sulfate (SLS) nasal solution (positive control) and the cavities of the untreated animals.

**Figure 6:**
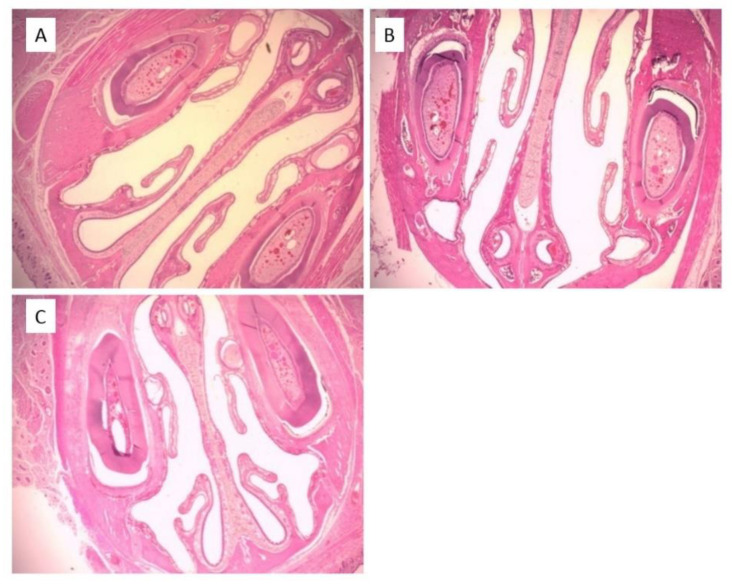
Representative micrographs of nasal cavities excised from rats (**A**) received no treatment (**B**) treated nasally with nasal nanovesicular carrier (NVS), and (**C**) treated nasally with normal saline (NS) (10×).

The micrographs of mucosa of animals treated with NVS and NS carriers showed intact mucosal epithelium, empty lumen and no infiltration of inflammatory cells (Figure 6B,C). Overall, there was no evidence of inflammation or irritation. Turbinate bone integrity was preserved. Epithelium was normal with no evidence of erosion or ulceration, and ciliated epithelium was intact. No pathological findings were observed in the histopathological analysis of the nasal cavities excised from rats treated with NVS or NS. The data indicate that sub-chronic administration of NVS has a similar effect on the nasal mucosa to that of NS. The micrographs of mucosa of animals treated with NVS and NS carriers were similar to those of the untreated animals (Figure 6A). On the other hand, proteinaceous material in the lumen and focal aggregations of neutrophils were observed in the mucosa of animals treated with SLS (micrograph not shown). Such observations suggest that the new carrier is locally safe during the tested period.

## 4. Discussion

In this work, we investigated the possibility of reducing strong pain by administration of non-controlled analgesic drugs nasally with enhanced delivery.

Strong pain is difficult to manage, which may negatively impact the patients’ quality of life. The chosen analgesic treatment must have a rapid onset of action. For such cases, including cancer-related breakthrough pain, the most common approach for management of pain is the oral administration of immediate-release opioids. In this type of administration, the active agent first appears in the blood 30 min following treatment [30]. In a pilot clinical study evaluating the analgesic effect of orally administrated immediate release morphine, oxycodone and hydromorphone to hospice patients, the average time to pain relief was greater than 30 min, while the average duration of pain in these patients was 35 min [31].

Nasal administration of opioids was investigated in some studies to obtain a rapid onset of action for the management of breakthrough pain in cancer patients. Pavis et al. [2] suggested a nasal morphine–chitosan formulation as a tolerable approach that may lead to quick pain relief. Fourteen patients, experiencing 20 breakthrough pain episodes, received the treatment at a morphine dose of 5–80 mg/kg. The results indicated that the formulation was acceptable to patients and had an onset of pain relief within 5 min after dosing. Noteworthy, sedation, a common side effect of strong opioids, was reported in 16 episodes.

In another non-randomized, open-label, uncontrolled study, the pharmacokinetics, safety and efficacy of a morphine gluconate nasal formulation, containing oleic acid and polysorbate 20, were evaluated in 11 cancer patients with breakthrough pain. A C_max_ of 64 ng/mL was achieved after 0.36 h, and an onset of meaningful effect of 6.8 min was reported by five patients. Nasal irritation (rhinitis) was reported by nine patients (81.8%). This local side effect could be due to the use of a surfactant (Polysorbate 20) [8].

Nasal fentanyl citrate is another strong opioid administration studied in a clinical non-controlled study on 12 cancer hospice in-patients with breakthrough pain. Before and after nasal treatment with 20 μg fentanyl citrate in aqueous solution, the participants were asked to rate their pain using a visual analogue scale (VAS). Thirty percent of the patients reported a reduction in their pain score within 5 min and 60% within 10 min [9].

Common side effects of strong opioids, including physical dependence and tolerance, raise clinical concerns and may prevent their prescription for pain management [12]. Therefore, new treatment strategies are required for rapid pain relief based on drugs with a better safety profile.

Here, we present results on nanovesicular analgesic systems (NVS) for nasal administration containing the non-controlled analgesic drugs Tramadol, Ketoprofen and Butorphanol.

These nasal delivery systems contain phospholipid multilamellar soft vesicles. The nasal vesicular carrier not containing the drug was shown to have a mean vesicular size of 480 nm. Incorporation of Tramadol and Butorphanol did not affect the vesicular size (509 and 453 nm). On the other hand, KET-NVS exhibited a 5-fold lower mean size of less than 100 nm. Further studies are needed to understand the effect of this active molecule on the vesicular size. The fluidization of the vesicle lamellar bilayers is caused by the presence of propylene glycol, as we have shown in a previous publication [23].

CLSM micrographs of the carrier loaded with FITC and R6G indicated the ability of the nanovesicles to entrap active molecules with various properties.

The pharmacokinetic results show that the concentration of Tramadol in rats’ plasma and brain increased rapidly after administration, reaching a peak value 10 min after administration, with a C_max_ of 2 to 5 folds greater that of the oral (TRA PO) or nasal non-vesicular (NV) treatments, respectively. In the case of Ketoprofen, the peak of the drug level in plasma was achieved 10 min after nasal administration in NVS. The C_max_ was three-fold higher relative to oral Ketoprofen (KET PO). A bioavailability of 1.56 relative to oral administration was calculated.

These results are sustained by the analgesic effect evaluated in an animal model for pain. Ten minutes after treatment with Tramadol and Butorphanol nanovesicular systems, the numbers of writhes were significantly reduced, showing MPE values of 63.1 and 62.6% for the Tramadol and Butorphanol systems, respectively. On the other hand, the same drugs administrated either orally or nasally in other carriers than NVS yielded values of less than 40% at all the tested time points. For Ketoprofen, 30 min post drug administration in NVS, an MPE of 67.0% was measured, and only 28.9% for the oral treatment. These results are supported by the superior drug plasma levels reached shortly after treatment. In further studies, it will be interesting to examine the analgesic effect of Ketoprofen NVS 10 min following administration. We think that nasal administration of analgesics in NVS led to quick and improved pain relief in mice, which involved systemic and nose to brain delivery.

The ability of the nasal nanovesicular carrier to enhance the delivery of analgesic drugs is attributed to the softness of the phospholipid vesicles. Such vesicles are, in general, formed in the presence of a bilayer fluidizing agent [25]. Ethosome was the first described phospholipid vesicle with bilayers fluidized by ethanol that was used for dermal delivery [32,33]. In a further work, we designed a nanovesicular carrier for nasal drug administration containing soft phospholipid vesicles in the presence of ethanol. This carrier enhanced the in vivo penetration of FITC, FITC–bacitracin and Rhodamine B into rats’ nasal mucosa, supporting the presumption that fluidity of the vesicles contributes to delivery enhancement [21]. This carrier improved delivery to the brain and to the systemic circulation as well as the pharmacodynamic effect of Buspirone and Glatiramer acetate [20,24]. In other experiments, the above discussed carrier effect on the analgesic effect of Tramadol was tested in animal pain models [19,21]. Although the analgesic effect with this system can be considered comparable to the results obtained in the present work, the use of ethanol can be a limitation for certain ethnic populations. This is not the case for the systems containing ethanol applied on the skin.

In a different work, we used propylene glycol to obtain phospholipid soft vesicles in Phospholipid Magnesome, a nanocarrier for direct nose to brain delivery of peptides, proteins and small molecules. The obtained data indicated its ability to enhance the nasal delivery of R6G, Insulin, and Epidermal growth factor (EGF) to various brain regions. Further, the antinociceptive effect of Oxytocin, a central-acting peptide, was improved in pain mice when administrated in Phospholipid Magnesome in comparison with nasal liposome, the nonvesicular system and a water solution.

The use of glycol was extended to glycerol by the group of Fadda to generate glycerosomes [34]. Recently, the potential use of glycerosomes to enhance the nasal delivery of Lacidipine, an anti-hypertensive agent, was investigated. The authors reported that nasal administration of this system, to rats with methylprednisolone acetate-induced hypertension, reduced their blood pressure values to normal [35].

The chosen drugs investigated here include the central-acting analgesic, Tramadol. During the last two decades, Tramadol prescription has increased significantly. This could be due to the drug’s good safety profile compared to other opioids such as Morphine. In addition, being a non-controlled drug in many countries, Tramadol has a low potential to cause addiction and abuse [36,37]. Currently, Tramadol is mostly administrated orally. The drug crosses the blood–brain barrier [38] and is almost completely absorbed following oral administration, reaching a peak plasma concentration within 1–2 h. Such a long T_max_ results in a relatively slow onset of action, which exceeds one hour, with a peak effect at 2–4 h [39]. Nasal administration in the nanovesicular carrier allows direct nose to brain delivery of the drug with the advantage of a short onset of action. The treatment is expected to act rapidly after administration, thus achieving a better patient compliance.

Butorphanol is another central-acting synthetic non-controlled opioid investigated in the present work. Results show a two-fold increase in analgesia in animals following nasal administration of this drug in NVS as compared to water solution.

Ketoprofen, the third analgesic drug studied here, is a systemic-acting nonsteroidal anti-inflammatory drug (NSAID) [40]. The drug was shown to be effective and well tolerated in hospitalized patients with cancer pain with comparable effect to that of the Aspirin and Codeine combination [41]. However, the oral route may be troublesome in advanced cancer patients due to worsening symptoms and physical decline necessitating an alternative option to this route. In another study, Moselli et al. [42] investigated the effect of long-term continuous subcutaneous infusion of Ketoprofen combined with Morphine in patients with cancer pain. The results showed that the treatment approach is feasible, safe, and effective for management of cancer pain. Nasal administration of Ketoprofen is expected to act rapidly and noninvasively to help cancer patients to control pain. To the best of our knowledge, this is the first work on the analgesic effect and the pharmacokinetic profile of Ketoprofen following its nasal administration.

Assessment of safety of nasally administrated systems is a key parameter to avoid irritation and toxicity in the nasal cavity. The local safety of the nanovesicular carrier tested on rats following sub-chronic administration shows no pathological changes or inflammation signs in the treated nasal mucosa, suggesting safety of the carrier for the tested period.

## 5. Conclusions

Nasal administration of Tramadol, Ketoprofen, and Butorphanol, in an nanovesicular system (NVS) containing soft phospholipid vesicles, led to a quick onset and improved analgesic effect in an animal model of pain. The carrier was found to be safe on the nasal mucosa for the tested period.

The findings of this work support the hypothesis that nasal carrier containing phospholipid soft nanovesicles enables short onset and enhanced analgesia using central- and systemic-acting non-controlled drugs. The data encourage clinical studies in this field. This new approach suggests that nasal delivery of non-controlled drugs in soft nanovesicles may open the way for better and noninvasive treatment of severe pain.

## Figures and Tables

**Table 1:** Physicochemical and pharmacological properties of the investigated drugs for pain management.

Drug Name	Molecular Weight (g/mol) *	LogP *	Pharmacological Classification	Pharmacological Indication for Pain Management	Available Products for Nasal Administration
Ketoprofen	254.3	3.12	Nonsteroidal anti-inflammatory drug (NSAIDs)	Relief of moderate pain	--
Butorphanol tartrate	477.5	3.3	Non-controlled synthetic opioid analgesic	Relief of moderate to severe acute pain	Stadol NS^®^
Tramadol hydrochloride	263.4	1.34	Non-controlled synthetic opioid analgesic	Relief of moderate to severe pain	--

* https://go.drugbank.com/ (accessed on 16 March 2021).

**Table 2:** Experimental parameters in testing analgesia following treatment with drugs in NVS, nasal water solution (WS), nasal non-vesicular system (NV) and oral preparation (PO) in animal model.

Drug	Drug Concentration in Systems (% *w*/*w*)	Administrated Drug Dose (mg/kg)	Testing Time after Treatment (min)
Ketoprofen	3	11	30
Butorphanol Tartrate	0.04	0.15	10
Tramadol HCl	0.9	5	10, 60, 180

**Table 3:** Compositions and properties of nanovesicular carrier and selected NVS containing non-controlled drugs for pain management.

System	Nasal Nanovesicular Carrier	Ketoprofen NVS	Butorphanol NVS	Tramadol NVS
Components, % *w*/*w*	PL:PG:DDW 2:30:68	KET:PL:PG:DDW 20:2:30:48	BUT:PL:PG:DDW 1:2:20:77	TRA:PL:PG:DDW 10:3:20:67
Organoleptic properties	Homogenous, semitransparent, whitish liquid	Homogenous, clear and slightly yellowish liquid	Homogenous, semitransparent, whitish liquid	Homogenous milky, non transparent, whitish liquid
Vesicles structure by TEM	Multilamellar vesicles	--	Multilamellar vesicles	--
pH	5.67 ± 0.12	5.54 ± 0.06	5.79 ± 0.03	5.00 ± 0.04
Vesicle size Diameter, nm	480.1 ± 170.1	99.41 ± 5.74	508.9 ± 70.2	453.1 ± 33.0
PDI	0.37 ± 0.12	0.13 ± 0.02	0.33 ± 0.10	0.19 ± 0.05

PL—Phospholipon 90G; PG—Propylene glycol; DDW—Double distilled water; KET—Ketoprofen; BUT—Butorphanol tartrate; TRA—Tramadol HCl; PDI—polydispersity index.

**Table 4:** Pharmacokinetic (pK) parameters for Ketoprofen following nasal administration in the nanovesicular system (KET NVS) as compared to oral preparation (KET PO), (Mean ± SD), (Values obtained using the NCOMP program).

PK Parameters	KET NVS	KET PO
T_max_ (min)	10.0 ± 0.0	30.0 ± 0.0
C_max_ (µg/mL)	43.65 ± 8.30	13.68 ± 5.91
AUC_0–5h_ (µg × min/mL)	4130.38 ± 730.58	2642.14 ± 1153.73
F_(relative to PO)_	1.56	

**Table 5:** Pharmacokinetic (pK) parameters for Tramadol in brain and plasma following administration in the nanovesicular system (NVS), Nasal non-vesicular system (NV) and Oral preparation (PO).

pK Parameter	Brain			Plasma		
	NVS	NV	PO	NVS	NV	PO
T_max_ (min)	10.0	10.0	60.0	10.0	10.0	60.0
C_max_ (µg/g)-for brain (ng/mL)-for plasma	3.46	2.08	0.73	743.6	529.3	329.5
AUC_0–240_ (µg × min/g)-for brain (ng × min/mL)-for plasma	361.80	195.90	168.43	17,577.0	8002.1	9660.7
F _relative to PO_	2.15	1.16		1.82	0.83	

**Table 6:** MPE% following administration of Tramadol nasal nanovesicular system (TRA NVS) to mice as compared to Nasal non-vesicular system (TRA NV) and Oral administration (TRA PO), each at a drug dose of 5 mg/kg, at various time points prior to pain induction.

Time (min)	NVS	NV	PO
	MPE%		
**10**	59.7	31.6	19.8
**60**	59.2	29.2	36.8
**180**	63.1	30.4	38.2

## Data Availability

Not applicable.
